# Fasting during Ramadan and acute kidney injury (AKI): a retrospective, propensity matched cohort study

**DOI:** 10.1186/s12882-022-02674-1

**Published:** 2022-02-07

**Authors:** Numan A. AlAbdan, Omar A. Almohammed, Maryam S. Altukhaim, Mahfooz A. Farooqui, Mubarak I. Abdalla, Hazza Q. Al Otaibi, Norah R. Alshuraym, Shahad N. Alghusun, Lama H. Alotaibi, Abdullah A. Alsayyari

**Affiliations:** 1 Pharmaceutical Care Department, King Abdulaziz Medical City–Ministry of National Guard Health Affaire, Riyadh, Saudi Arabia; 2 King Abdullah International Medical Research Center, Riyadh, Saudi Arabia; 3 King Saud Bin Abdulaziz University for Health Sciences, College of Pharmacy, Riyadh, Saudi Arabia; 4 Department of Clinical Pharmacy, College of Pharmacy, King Saud University, Riyadh, Saudi Arabia; 5 Pharmacoeconomics Research Unit, College of Pharmacy, King Saud University, Riyadh, Saudi Arabia; 6 Division of Nephrology and Renal Transplantation, Department of Medicine, King Abdulaziz Medical City–Ministry of National Guard Health Affaire, Riyadh, Saudi Arabia; 7 College of Medicine, King Saud Bin Abdulaziz University for Health Sciences, Riyadh, Saudi Arabia

**Keywords:** Acute kidney injury, Kidney disease, Fasting, Ramadan, Serum creatinine

## Abstract

**Background:**

During the month of Ramadan, Muslims abstain from daytime consumption of fluids and foods, although some high-risk individuals are exempt. Because fasting's effects on the risk of acute kidney injury (AKI) have not been established, this study assesses the relationship between fasting and risk of AKI and identifies patients at high risk.

**Methods:**

A single-center, retrospective, propensity-score matched, cohort study was conducted with data collected from adult patients admitted to the emergency room during Ramadan and the following month over two consecutive years (2016 and 2017). AKI was diagnosed based on the 2012 definition from the Kidney Disease: Improving Global Outcomes clinical practice guideline. Multivariable logistic regression analyses were used to examine the correlation and measure the effect of fasting on the incidence of AKI, and assess the effect of different variables on the incidence of AKI between the matching cohorts.

**Results:**

A total of 1199 patients were included; after matching, each cohort had 499 patients. In the fasting cohort, the incidence of AKI and the risk of developing AKI were significantly lower (adjusted odds ratio (AOR) 0.65;95% confidence interval (CI) 0.44–0.98). The most indicative risk factors for AKI were hypertension (AOR 2.17; 95% CI 1.48–3.18), history of AKI (AOR 5.05; 95% CI 3.46–7.39), and liver cirrhosis (AOR 3.01; 95% CI 1.04–8.70). Patients with these factors or most other comorbidities in the fasting cohort had a lower risk of AKI as compared with their nonfasting counterparts.

**Conclusion:**

The data show a strong reduction in the risk of developing AKI as a benefit of fasting, particularly in patients with comorbid conditions. Therefore, most patients with comorbid conditions are not harmed from fasting during Ramadan. However, larger prospective studies are needed to investigate the benefit of fasting in reducing the risk of developing AKI.

## Background

In Islam, fasting during the lunar month of Ramadan is obligatory [1]. When fasting, Muslims abstain from sex and from consuming fluids, foods, and other things that enter the body and can be absorbed, such as oral medications, from dawn until sunset. However, other routes of administration are exempt, because they do not nullify fasting, with the exception of intravenous feeding or injections [1, 2]. For some individuals, fasting might be difficult or even harmful to their health. Individuals in whom the imposed difficulty is temporary, such as during traveling, in those with acute illness, and in women who are pregnant, menstruating, or having postpartum bleeding, are excused from fasting and can compensate for the missed days at a later time. However, individuals unable to fast because of a persistent condition, such as severe chronic illness that is not expected to be cured or controlled, are exempt from fasting obligations [3].

According to the 2012 Kidney Disease: Improving Global Outcomes (KDIGO) clinical practice guideline for acute kidney injury (AKI), AKI is defined as an abrupt decrease in kidney function that includes, but is not limited to, acute renal failure [4]. The diagnosis and staging of AKI is based on serum creatinine (SCr) level and urine output [4]. Identifying risk factors for developing AKI can help in prevention, including advanced age, chronic kidney disease (CKD), and other chronic comorbidities. In addition, nephrotoxic substances such as contrast media and some medications (e.g., angiotensin-converting enzyme inhibitors [ACE-Is] and angiotensin receptor blockers [ARBs]) are associated with AKI [5, 6].

Recent studies investigated the relationship between fasting during Ramadan and kidney function with AKI. One study included patients with CKD and evaluated them for AKI. The number of days patients fasted and hypertension were predictive of AKI; patients with AKI fasted fewer days and were more likely to have hypertension [7]. Another study evaluated the impact of abstaining from drinking water during fasting Ramadan on AKI and found that thirst did not increase the risk of AKI for patients with normal kidney function [8].

Repeat subclinical AKI from chronic volume depletion has been proposed as predisposing patients to CKD [9, 10], because volume depletion is associated with CKD prevalence, particularly when coupled with situations that increase water loss such as high temperature and intensive physical activities [11]. However, there is no evidence demonstrating the link between repeated volume depletion and the development of CKD. Even among marathon runners who have a high risk of AKI, there is no evidence of increased risk of CKD [12, 13]. However, dehydration has multiple effects on the kidney and is linked to AKI [14], also it might lead to CKD [15]. Therefore, in patients with or at increased risk of CKD, hydration is recommended for preserving kidney function [16]. In contrast, dietary restriction could be beneficial for the kidneys. For instance, dietary restriction had a significant nephroprotective effect in rats [17]. Although dietary restriction may offer some renal protection, it has not been clinically effective in preventing contrast-induced AKI at 48 h after percutaneous coronary intervention [18].

In-hospital mortality rates due to AKI are extremely high and persist after discharge [19]. In addition, the lack of effective treatment for AKI; the associated risk of long-term complications, such as the risk of developing CKD; and the overall high cost of managing AKI, especially for patients who require renal replacement therapy, indicate that the incidence of AKI can place a huge burden on these patients' quality of life and the health care system [20]. Previous studies investigated various AKI risk factors including drug-induced AKI, CKD, diabetes, hypertension, and so forth [21–25]. However, only limited studies have evaluated the relationship between fasting and the incidence of AKI. Therefore, we conducted this study to assess the relationship between fasting and the risk of developing AKI and to identify patients at high risk of developing AKI.

## Methods

### Study design and setting

We conducted a retrospective cohort study to evaluate the association between fasting and the incidence of AKI and to identify patients at high risk for developing AKI while fasting. The study was conducted using data from King Abdulaziz Medical City (KAMC). Data for patients admitted through the emergency room (ER) to KAMC in Ramadan and the following lunar month (as this month most resemble the climate of Ramadan) were reviewed; eligible patients were included in the fasting and nonfasting cohorts, respectively.

### Subjects and data collection

All adult patients admitted through the ER with stable baseline SCr level before admission and who were not religiously excused from fasting because of any severe chronic medical condition were included. Patients were excluded if they had an end-stage renal disease or were undergoing cancer therapy. In addition, patients were excluded if they did not have a baseline SCr, had incomplete medical records, or had an elective admission.

Data were collected retrospectively from the electronic medical records of patients admitted to the ER during Ramadan in 2016 and 2017 and from a cohort of patients admitted during the following lunar month (Shawwal) in 2016 and 2017. For the fasting cohort, we collected data from patients who were admitted during the month of Ramadan, which occurred between June 6 and July 5, 2016, and between May 27 and June 24, 2017. For the nonfasting cohort, data were collected for patients who were admitted during the following lunar month, which occurred between July 6 and August 3, 2016, and between June 25 and July 23, 2017. Data included patient demographics, comorbid conditions, vital signs, lab results, prescribed medications, and incidence of new AKI, defined as an increase in SCr of ≥ 0.3 mg/dL (26.5 µmol/L) within 48 h or an increase in SCr by 1.5 times of baseline that is known or presumed to have happened within the past 7 days [4].

### Ethical considerations

We ensured patient confidentiality by assigning a study ID number that was used in the data collection form, and all collected data were kept in a secure location. The Institutional Review Board at King Abdullah International Medical Research Center reviewed and approved the study protocol (RC19/136/R) and waived the need for written informed consent.

### Statistical analysis

To describe the study population, we used descriptive statistics including mean (± SD) for continuous variables and frequencies with percentages for categorical variables. Patients' baseline characteristics (age and gender), Charlson Comorbidity Index (CCI), comorbid conditions (such as CKD, diabetes, hypertension, history of AKI (defined as any reported AKI insult before the current admission), cardiomyopathy, etc.), baseline clinical parameters (SCr and albumin levels), and medications used before admission were compared between the two cohorts using *t* test for continuous variables and chi-square test for categorical data. To make the two cohorts comparable to each other, the cohorts were matched in terms of baseline characteristics, CCI, comorbid conditions, and baseline clinical parameters, only. The cohorts were matched using propensity-score (PS) matching with the greedy nearest neighborhood matching method with a caliber of 0.2 SD for the logit of the estimated PS.

Univariable logistic regression analysis was used to examine the correlation and measure the effect of fasting only on the incidence of AKI; and the result was reported in the unadjusted analysis as crude odds ratio (COR) with 95% confidence interval (95%CI). Then, multilevel logistic regression model was used to assess the association and measure the effect of baseline characteristics, CCI, comorbid conditions, baseline clinical parameters, and medications used before admission on the incidence of AKI while controlling for the effect of fasting as a grouping variable in the multilevel model; results were reported in the unadjusted analysis. Then, backward-stepwise multivariable logistic regression, with *p* < 0.1 for keeping variables in the model, was used to examine important predictors for the incidence of AKI; and results were reported in the adjusted analysis as adjusted OR (AOR) with 95%CI. A *p-*value < 0.05 was considered statistically significant. The two cohorts were matched using the Proc PSMATCH procedure for propensity score analysis using SAS software [26, 27]. All data were analyzed using SAS software, version 9.4 (SAS Institute Inc., Cary, NC).

## Results

### Baseline characteristics

A total of 1199 patients were included in the study but the two cohorts were not balanced in term of the baseline characteristics. After matching, we ended-up with a set of 499 patients in each cohort, with the fasting and nonfasting cohorts totaling to 998 patients. Before matching, the nonfasting cohort were significantly older in age and had a higher proportion of patients with a CCI score of more than four points compared to the fasting cohort. In addition, the nonfasting cohort had a higher proportion of patients with different comorbidities across the board. However, after matching all differences between the two cohorts on the previously mentioned confounding variables became insignificant, as presented in the nonmatched and matched columns of Table 1. Moreover, there was no significant difference in baseline clinical parameters between the fasting and nonfasting cohorts before and after matching (Table 1). However, there was a significant difference in medications prescribed before admission; Table 1 shows that even after matching for all other baseline characteristics, the nonfasting cohort had a significantly greater usage of nephrotoxic medications.

**Table 1:** Patient characteristics, baseline comorbidities, and medications used before admission (before and after matching)

Patients Characteristic	Nonmatched cohorts				Matched cohorts			
	**Overall** **(*N* = 1199)**	**Nonfasting** **(*n* = 599)**	**Fasting** **(*n* = 600)**	*p* value^*^	**Overall** **(*N* = 998)**	**Nonfasting** **(*n* = 499)**	**Fasting** **(*n* = 499)**	***p* value**
Age in years, mean (SD)	51.3 (21.3)	53.3 (22.2)	49.3 (20.1)	**0.001**	50.1 (21.4)	50.4 (22.2)	49.7 (20.4)	0.612
Age > 60 years	498 (40.8)	270 (45.1)	219 (36.5)	**0.002**	387 (38.8)	194 (38.9)	193 (38.7)	0.948
Gender (female)	543 (44.5)	262 (43.7)	272 (45.3)	0.578	464 (46.5)	222 (44.5)	242 (48.5)	0.204
BMI (kg/m^2^), mean (SD)	27.6 (6.0)	27.61 (6.1)	27.58 (5.9)	0.926	27.5 (6.1)	27.5 (6.1)	27.7 (6.1)	0.539
CCI > 4	482 (40.2)	262 (43.7)	220 (36.7)	**0.013**	376 (37.7)	188 (37.7)	188 (37.7)	1.000
Comorbidities								
Hypertension	528 (44.0)	288 (48.0)	240 (40.0)	**0.005**	411 (41.2)	208 (41.7)	203 (40.7)	0.748
Diabetes mellitus	518 (43.2)	274 (45.7)	244 (40.7)	0.076	401 (40.2)	208 (41.7)	193 (38.7)	0.333
History of AKI	306 (25.5)	164 (27.4)	142 (23.7)	0.140	239 (24.0)	117 (23.5)	122 (24.5)	0.711
Cardiomyopathy	144 (12.0)	94 (15.7)	50 (8.3)	** < 0.001**	120 (12.0)	70 (14.0)	50 (10.0)	0.052
Anemia	140 (11.7)	91 (15.2)	49 (8.2)	** < 0.001**	101 (10.1)	52 (10.4)	49 (9.8)	0.753
Ischemic heart diseases	139 (11.6)	96 (16.0)	43 (7.2)	** < 0.001**	94 (10.2)	51 (10.2)	43 (8.6)	0.386
Heart failure	132 (11.0)	67 (11.2)	65 (10.8)	0.845	112 (11.2)	48 (9.6)	64 (12.8)	0.109
Stroke	122 (10.2)	70 (11.7)	52 (8.7)	0.084	94 (9.4)	48 (9.6)	46 (9.2)	0.828
Asthma	113 (9.4)	63 (10.5)	50 (8.33)	0.196	94 (9.4)	49 (9.8)	45 (9.0)	0.665
Dyslipidemia	78 (6.5)	55 (9.2)	23 (3.8)	** < 0.001**	47 (4.7)	24 (4.8)	23 (4.6)	0.881
Chronic kidney disease	82 (6.0)	46 (7.7)	36 (6.0)	0.249	61 (6.1)	28 (5.6)	33 (6.6)	0.509
Hypothyroidism	70 (5.5)	42 (7.0)	28 (4.7)	0.083	49 (4.9)	30 (6.0)	19 (3.8)	0.107
Chronic obstructive pulmonary disease	58 (4.8)	30 (5.0)	28 (4.7)	0.782	47 (4.7)	19 (3.8)	28 (5.6)	0.179
Seizure	38 (3.2)	22 (3.7)	16 (2.7)	0.320	28 (2.8)	14 (2.8)	14 (2.8)	1.000
Liver cirrhosis	27 (2.3)	15 (2.5)	12 (2.0)	0.556	19 (1.9)	9 (1.8)	10 (2.0)	0.817
Kidney transplant	21 (1.8)	7 (1.2)	14 (2.3)	0.124	19 (1.9)	7 (1.4)	12 (2.4)	0.247
History of cancer	15 (1.3)	12 (2.0)	3 (0.5)	**0.019**	4 (0.4)	1 (0.2)	3 (0.6)	0.316
Liver transplant	14 (1.2)	7 (1.2)	7 (1.2)	0.998	11 (1.1)	4 (0.8)	7 (1.4)	0.363
History of VTE	9 (0.8)	5 (0.8)	4 (0.7)	0.736	5 (0.5)	3 (0.6)	2 (0.4)	0.313
Hepatitis B	9 (0.7)	8 (1.3)	1 (0.2)	**0.021**	2 (0.2)	1 (0.2)	1 (0.2)	1.000
Hepatitis C	7 (0.6)	6 (1.0)	1 (0.2)	0.058	2 (0.2)	1 (0.2)	1 (0.2)	1.000
Glomerulonephritis	4 (0.3)	2 (0.3)	2 (0.3)	0.999	3 (0.3)	1 (0.2)	2 (0.4)	0.563
Clinical parameter								
Baseline SCr level (µmol/L)				0.058				0.400
< 60	232 (19.4)	104 (17.4)	128 (21.3)		207 (20.8)	95 (19.1)	115 (23.1)	
60–110	854 (71.2)	429 (71.6)	425 (70.8)		709 (71.0)	361 (72.3)	345 (69.1)	
> 110	113 (9.4)	66 (11.0)	47 (7.8)		82 (8.2)	43 (8.6)	39 (7.8)	
Baseline eGFR level (mL/min)				0.095				0.792
≥ 60	1044 (87.1)	513 (85.6)	531 (88.5)		883 (88.5)	438 (87.8)	444 (89.0)	
45–59	94 (7.8)	46 (7.7)	48 (8.0)		67 (6.7)	34 (6.8)	34 (6.8)	
30–44	38 (3.2)	25 (4.2)	13 (2.2)		29 (2.9)	16 (3.2)	13 (2.6)	
15–29	18 (1.5)	13 (2.2)	5 (0.8)		14 (1.4)	9 (1.8)	5 (1.0)	
< 15	5 (0.4)	2 (0.3)	3 (0.5)		5 (0.5)	2 (0.4)	3 (0.6)	
Baseline albumin level (g/L)				0.340				0.607
Hypo < 35	903 (75.3)	444 (74.1)	459 (76.5)		756 (75.8)	379 (75.9)	372 (74.6)	
Normal ≥ 35	296 (24.7)	155 (25.9)	141 (23.5)		242 (24.2)	120 (24.1)	127 (25.4)	
Medications used before admission								
PPIs	473 (39.5)	282 (47.1)	191 (31.8)	** < 0.001**	385 (38.6)	218 (43.7)	166 (33.3)	** < 0.001**
ARBs	264 (22.0)	236 (39.4)	28 (4.7)	** < 0.001**	211 (21.1)	183 (36.7)	27 (5.4)	** < 0.001**
Diuretics	209 (17.4)	136 (22.7)	73 (12.2)	** < 0.001**	159 (15.9)	92 (18.4)	67 (13.4)	**0.030**
ACE-Is	126 (10.5)	104 (17.4)	22 (3.7)	** < 0.001**	91 (9.1)	73 (14.6)	18 (3.6)	** < 0.001**
NSAIDs	117 (9.8)	83 (13.9)	34 (5.7)	** < 0.001**	97 (9.7)	72 (14.4)	25 (5.0)	** < 0.001**
Immunosuppressive medications	26 (2.2)	9 (1.5)	17 (2.8)	0.114	21 (2.1)	7 (1.4)	15 (3.0)	0.085

Results are presented as frequency (%) unless otherwise indicated

^*^ *p* value from *t* test or chi-square test; values < 0.05 were considered statistically significant

Numbers in bold indicate significant results

Abbreviations: *SD* standard deviation, *BMI* body mass index, *CCI* Charlson Comorbidity Index, *AKI* acute kidney injury, *VTE* venous thromboembolism, *SCr* serum creatinine, *eGFR* estimated glomerular filtration rate, *PPIs* proton-pump inhibitors, *ACE-Is* angiotensin-converting enzyme inhibitors, *ARBs* angiotensin receptor blockers, *NSAIDs* nonsteroidal anti-inflammatory drugs

### Incidence of AKI

The incidence of AKI was significantly higher in the nonfasting cohort (20.4%) compared with the fasting cohort (13.4%), which was statistically significant even after adjustment for other important factors in the multivariable logistic regression (AOR 0.66; 95% CI 0.44–0.98), as shown in Table 2. This means that fasting offers some protection against the incidence of AKI, with fasting patients having a 35% lower odds of AKI as compared with their nonfasting counterparts.

**Table 2:** Assessment of factors associated with incidence of acute kidney injury (AKI) in the matched cohorts

Variable	Category	Nonfasting (Non-Ramadan)		Fasting (Ramadan)		Unadjusted analysis	Adjusted analysis
		**No AKI**	**AKI**	**No AKI**	**AKI**	**COR (95%CI)**^**a**^	**AOR**^**b**^** (95%CI)**
Ramadan (fasting)		397 (79.6)	102 (20.4)	432 (86.6)	67 (13.4)	**0.60 (0.43–0.85)**	**0.65 (0.44 – 0.98)**
Age > 60 years	No	261 (52.3)	44 (8.8)	282 (56.5)	24 (4.8)	1.0	—
	Yes	136 (27.3)	58 (11.6)	150 (30.1)	43 (8.6)	**2.85 (2.02–4.00)**	—
CCI > 4	No	277 (55.5)	34 (6.8)	290 (58.1)	21 (4.2)	1.0	—
	Yes	120 (24.1)	68 (13.6)	142 (28.5)	46 (9.2)	**4.56 (3.19–6.50)**	—
Gender	Male	222 (44.5)	55 (11.0)	216 (43.3)	41 (8.2)	1.0	—
	Female	175 (35.1)	47 (9.4)	216 (43.3)	26 (5.2)	0.87 (0.62–1.21)	—
Comorbidities							
Hypertension	No	257 (51.5)	34 (6.8)	273 (54.7)	23 (4.6)	1.0	1.0
	Yes	140 (28.1)	68 (13.6)	159 (31.9)	44 (8.8)	**3.5 (2.47–4.97)**	**2.17 (1.48–3.18)**
Diabetes mellitus	No	249 (49.9)	42 (8.4)	276 (55.3)	30 (6.0)	1.0	—
	Yes	148 (29.7)	60 (12.0)	156 (31.3)	37 (7.4)	**2.30 (1.65–3.23)**	—
History of AKI	No	340 (68.1)	42 (8.4)	344 (68.9)	33 (6.6)	1.0	1.0
	Yes	57 (11.4)	60 (12.0)	88 (17.6)	34 (6.8)	**6.12 (4.28–8.74)**	**5.05 (3.46–7.39)**
Cardiomyopathy	No	349 (70.0)	80 (16.0)	391 (78.4)	58 (11.6)	1.0	—
	Yes	48 (9.6)	22 (4.4)	41 (8.2)	9 (1.8)	**1.80 (1.14–2.82)**	—
Anemia	No	357 (71.6)	90 (18.0)	390 (78.2)	60 (12.0)	1.0	—
	Yes	40 (8.0)	12 (2.4)	42 (8.4)	7 (1.4)	1.15 (0.67–1.95)	—
Ischemic heart diseases	No	363 (72.8)	85 (17.0)	401 (80.4)	55 (11.0)	1.0	—
	Yes	34 (6.8)	17 (3.4)	31 (6.2)	12 (2.4)	**2.40 (1.49–3.86)**	—
Heart failure	No	371 (74.4)	80 (16.0)	379 (76.0)	56 (11.2)	1.0	—
	Yes	26 (5.2)	22 (4.4)	53 (10.6)	11 (2.2)	**2.43 (1.55–3.82)**	—
Stroke	No	363 (72.8)	88 (17.6)	391 (78.4)	62 (12.4)	1.0	—
	Yes	34 (6.8)	14 (2.8)	41 (8.2)	5 (1.0)	1.27 (0.74–2.17)	—
Asthma	No	364 (73.0)	86 (17.2)	393 (78.8)	61 (12.2)	1.0	—
	Yes	33 (6.6)	16 (3.2)	39 (7.8)	6 (1.2)	1.56 (0.94–2.61)	—
Dyslipidemia	No	381 (76.4)	94 (18.8)	414 (83.0)	62 (12.4)	1.0	—
	Yes	16 (3.2)	8 (1.6)	18 (3.6)	5 (1.0)	**1.95 (1.01–3.80)**	—
Chronic kidney disease	No	384 (77.0)	87 (17.4)	411 (82.4)	55 (11.0)	1.0	—
	Yes	13 (2.6)	15 (3.0)	21 (4.2)	12 (2.4)	**4.65 (2.71–8.00)**	—
Hypothyroidism	No	375 (75.2)	94 (18.8)	420 (84.2)	60 (12.0)	1.0	—
	Yes	22 (4.4)	8 (1.6)	12 (2.4)	7 (1.4)	**2.17 (1.15**–**4.10)**	—
COPD	No	381 (76.4)	99 (19.8)	412 (82.6)	59 (11.8)	1.0	—
	Yes	16 (3.2)	3 (0.6)	20 (4.0)	8 (1.6)	1.62 (0.81–3.28)	—
Seizure	No	383 (76.8)	102 (20.4)	419 (84.0)	66 (13.2)	1.0	—
	Yes	14 (2.8)	0 (0.0)	13 (2.6)	1 (0.2)	0.18 (0.02–1.31)	—
Liver cirrhosis	No	394 (79.0)	96 (19.2)	425 (85.2)	64 (12.8)	1.0	1.0
	Yes	3 (0.6)	6 (1.2)	7 (1.4)	3 (0.6)	**4.77 (1.89–12.02)**	**3.01 (1.04–8.70)**
Kidney transplant	No	392 (78.6)	100 (20.0)	422 (84.6)	65 (13.0)	1.0	—
	Yes	5 (1.0)	2 (0.4)	10 (2.0)	2 (0.4)	1.42 (0.46–4.35)	—
History of cancer	No	396 (79.4)	102 (20.4)	430 (86.2)	66 (13.2)	1.0	—
	Yes	1 (0.2)	0 (0.0)	2 (0.4)	1 (0.2)	1.88 (0.19–18.43)	—
Liver transplant	No	393 (78.8)	102 (20.4)	426 (85.4)	66 (13.2)	1.0	—
	Yes	4 (0.8)	0 (0.0)	6 (1.2)	1 (0.2)	0.52 (0.07–4.12)	—
History of VTE	No	395 (79.2)	101 (20.2)	430 (86.2)	67 (13.4)	1.0	—
	Yes	2 (0.4)	0 (0.0)	2 (0.4)	0 (0.0)	1.17 (0.13–10.64)	—
Hepatitis B	No	396 (79.4)	102 (20.4)	431 (86.4)	67 (13.4)	1.0	—
	Yes	1 (0.2)	0 (0.0)	1 (0.2)	0 (0.0)	—	—
Hepatitis C	No	396 (79.4)	102 (20.4)	431 (86.4)	67 (13.4)	1.0	—
	Yes	1 (0.2)	0 (0.0)	1 (0.2)	0 (0.0)	—	—
Glomerulonephritis	No	397 (79.6)	101 (20.2)	430 (86.2)	67 (13.4)	1.0	—
	Yes	0 (0.0)	1 (0.2)	2 (0.4)	0 (0.0)	2.71 (0.24–30.61)	—
Clinical parameters							
Baseline SCr (µmol/L)	< 60	88 (17.6)	7 (1.4)	111 (22.2)	1 (0.2)	**0.18 (0.09–0.39)**	—
	60 – 110	287 (57.6)	74 (14.8)	295 (59.2)	53 (10.6)	1.0	—
	> 110	22 (4.4)	21 (4.2)	26 (5.2)	13 (2.6)	**3.27 (2.02–5.30)**	—
Baseline Albumin (g/L)	≥ 35	306 (61.3)	73 (14.6)	338 (67.8)	39 (7.8)	1.0	—
	< 35	91 (18.3)	29 (5.8)	94 (18.8)	28 (5.6)	**1.79 (1.25 – 2.56)**	—
Medications used before admission							
PPIs	No	230 (46.1)	51 (10.2)	292 (58.5)	40 (8.0)	1.0	—
	Yes	167 (33.5)	51 (10.2)	140 (28.1)	27 (5.4)	1.39 (0.99–1.95)	—
ARBs	No	261 (52.3)	55 (11.0)	411 (82.4)	60 (12.0)	1.0	1.0
	Yes	136 (27.3)	47 (9.4)	21 (4.2)	7 (1.4)	**1.74 (1.17–2.60)**	1.52 (0.97–2.36)
Diuretics	No	339 (67.9)	68 (13.6)	378 (75.8)	54 (10.8)	1.0	—
	Yes	58 (11.6)	34 (6.8)	54 (10.8)	13 (2.6)	**2.38 (1.61–3.53)**	—
ACE-Is	No	345 (69.1)	81 (16.2)	414 (83.0)	67 (13.4)	1.0	—
	Yes	52 (10.4)	21 (4.2)	18 (3.6)	0 (0.0)	1.32 (0.78–2.25)	—
NSAIDs	No	331 (66.3)	96 (19.2)	412 (82.6)	62 (12.4)	1.0	—
	Yes	66 (13.2)	6 (1.2)	20 (4.0)	5 (1.0)	0.52 (0.27–1.01)	—
Immunosuppressive medications	No	391 (78.4)	101 (20.2)	419 (84.0)	66 (13.2)	1.0	1
	Yes	6 (1.2)	1 (0.2)	13 (2.6)	1 (0.2)	0.55 (0.13–2.41)	0.23 (0.05–1.03)

Results are presented as frequency (%)

^a^The CORs are the crude odds ratio from the unadjusted analysis of the univariable logistic regression for Ramadan and the multilevel logistic regression analyses for other variables, while controlling for the effect of Ramadan in the model

^b^The AORs are the adjusted odds ratio from the backward-stepwise multivariable logistic regression model

Numbers in bold indicates significant results

Abbreviations: *AKI* acute kidney injury, *COR* crude odds ratio, *CI* confidence interval, *AOR* adjusted odds ratio, *CCI* Charlson Comorbidity Index, *AKI* acute kidney injury, *VTE* venous thromboembolism, *SCr* serum creatinine, *eGFR* estimated glomerular filtration rate, *PPIs* proton-pump inhibitors, *ACE-Is* angiotensin-converting enzyme inhibitors, *ARBs* angiotensin receptor blockers, *NSAIDs* nonsteroidal anti-inflammatory drugs

### Factors affecting the development of AKI

After controlling for the effect of fasting only on all other variables in the multilevel logistic regression, patients older than 60 years and those with a CCI score > 4 had a higher risk of AKI as compared with patients younger than 60 years (OR 2.85; 95% CI 2.02–4.00) and patients with a CCI score < 4 points (OR 4.56; 95% CI 3.19–6.50), respectively. However, after adjusting for the effect of all other variables in the multivariable logistic regression, both of these variables became insignificant. Table 2 presents the results from the crude and adjusted analyses.

Among all other comorbid conditions, hypertension, diabetes mellitus, history of AKI, dyslipidemia, cardiomyopathy, ischemic heart diseases, heart failure, CKD, hypothyroidism, and liver cirrhosis were all significant predictors of the incidence of AKI in the crude analysis. However, after controlling for the effect of all other variables in the adjusted analysis, having hypertension (AOR 2.17; 95% CI 1.48–3.18), a history of AKI (AOR 5.05; 95% CI 3.46–7.39), and liver cirrhosis (AOR 3.01; 95% CI 1.04–8.70) were the only variables that remained significant for predicting the incidence of AKI in both cohorts. Moreover, the odds of developing AKI when a patient had a history of AKI was about two times higher in the nonfasting (OR 8.52; 95% CI 5.25–13.82) as compared with the fasting cohort (OR 4.03; 95% CI 2.36–6.86).

Moreover, the data show that fasting confers some benefit for patient with comorbidities, as a lower proportion of patients with comorbidity developed AKI in the fasting cohort as compared with their nonfasting counterparts. For example, 32.7% of patients with hypertension had an AKI in the nonfasting cohort, as compared with 21.7% in the fasting cohort. This was similar for patients with diabetes mellitus, dyslipidemia, cardiomyopathy, and ischemic heart diseases. Moreover, that effect was even more pronounced in patients with heart failure (45.8% vs. 17.2%), CKD (53.6% vs. 36.4%), and liver cirrhosis (66.6% vs. 30.0%) for the nonfasting and fasting cohorts, respectively. However, among patients with hypothyroidism, the fasting cohort had a higher proportion of patients who developed AKI as compared with the nonfasting cohort (36.8% vs. 26.6%, respectively).

Considering the patients' baseline clinical parameters, a higher proportion of the fasting cohort (22.4%) had a baseline SCr less than 60 µmol/L as compared with the nonfasting cohort (19%), which was associated with lower risk of developing AKI in the unadjusted analysis (COR 0.18; 95% CI 0.09–0.39) but not the adjusted analysis. Moreover, the fasting cohort had fewer cases of AKI in each bracket of baseline SCr level than the nonfasting cohort did. Although there were more patients in the nonfasting cohort using nephrotoxic medications before admission, the use of nephrotoxic medications was not associated with an increased risk of developing AKI (Table 2).

## Discussion

This study aimed to investigate the relationship between fasting and the risk of AKI. The main question of this study was, “Does fasting during the month of Ramadan increase the risk of hospital admissions with AKI diagnosis?” Contrary to our expectations, fasting was in fact associated with fewer hospital admissions for AKI. We also investigated the risk factors for AKI to identify high-risk patients. From the multivariable logistic regression, we only found that patients with hypertension, a history of AKI, or liver cirrhosis had a higher odds of developing AKI, regardless of fasting status. However, the risk of AKI in patients with these risk factors was even higher in the nonfasting cohort as compared with the fasting cohort, particularly in nonfasting patients with a history of AKI, because their risk was half that of their matched fasting cohort. Patients with most of the comorbidities under investigation seem to benefit from fasting during Ramadan, as lower proportions of patients with comorbidities were admitted with a diagnosis of AKI in the fasting cohort as compared with their nonfasting counterparts.

Few studies have investigated the correlation between fasting during Ramadan and the risk of AKI. One study found no correlation between medication use and the development of AKI, similar to our findings. Their analysis also indicated that fasting for more days lowered the risk of developing AKI, whereas having hypertension increased the risk significantly, and unlike our study, patient age did not affect the risk [7]. In addition, fasting has been found to be beneficial in reducing patients' weight, waist circumference, and blood pressure and in providing an overall improvement in the lipid profile, which ultimately reduces the risk of cardiovascular diseases in fasting individuals [28–30].

Although the effect of fasting during Ramadan on kidney physiology is not well studied, the total amount of body fluid was found to be maintained during fasting [31]. In addition, previous studies evaluated the safety of fasting in patients with stages 2–4 CKD and found that fasting was well tolerated and offered other benefits in terms of weight loss and blood pressure reduction. One study found that fasting improved these patients' estimated glomerular filtration rate (eGFR), whereas another found no difference as compared with the control group [31, 32]. Fasting was also found to be safe in some patients on hemodialysis and in kidney transplant recipients [33–38]. Furthermore, one of these studies found that the change in eGFR was in favor of the fasting cohort during the first 5 years after transplantation [37]. Moreover, there was some controversy regarding the effect of fasting on SCr levels in patients with CKD [32, 39–41], but most of these studies found no significant difference. The only study to link fasting during Ramadan with worsening renal function and elevated SCr levels in patients with CKD had no control group and compared values only before, during, and after fasting for Ramadan [39].

We found that fasting could be beneficial in reducing the AKI risk and the odds of AKI among patients with predisposing risk factors for AKI. Hence, fasting can be a valuable intervention. Specifically, as demonstrated in previous studies, in patients with hypertension and other cardiovascular diseases, fasting is associated with benefits of lowering both weight and blood pressure as well as improvements in the lipid profile [28–30], and the findings of our study add to this evidence. The risk of AKI cannot always be controlled by pharmacologic interventions and intensive control, such as in the case of hypertension, in which intensive control with a systolic blood pressure goal of less than 120 mm Hg leads to an increased risk of AKI [42]. Therefore, fasting, or perhaps intermittent fasting, might be an effective nonpharmacologic intervention. However, further research is needed to confirm our findings, and the benefit of fasting should be investigated as compared with intermittent fasting.

One additional peculiar finding in our study was that fewer patients received treatment with nephrotoxic medications in the fasting cohort as compared with their nonfasting counterparts. Fewer than half the patients in the fasting cohort received prescribed medications, such as ACE-Is, ARBs, diuretics, and nonsteroidal anti-inflammatory drugs, even after matching for baseline characteristics. Therefore, future research should investigate and address the prescribing behaviors of physicians for these nephrotoxic medications during the month of Ramadan as compared with other months of the year as well as the reasoning behind this difference.

This is the first study to evaluate the correlation between fasting and risk of developing AKI using a matching control cohort. However, this was a retrospective observational study, which has some limitations, including the distinct difference in utilization for prescribed nephrotoxic medications between the two cohorts. However, a rigorous control for its effect was implied by matching on the baseline characteristics, then the use of prescribed nephrotoxic medications and the baseline characteristics were included in the multivariable logistic regression to control for their effect on the overall comparison, and none of these medications were found to be significant in the multivariable logistic regression. This was also a single-center study and conducted using chart review. Moreover, we were unable to include the full diagnosis criteria according to the KDIGO 2012 definition of AKI, as fasting and abstaining from drinking water will naturally drive the kidney to produce less urine. Therefore, it would be invalid to base the diagnosis of AKI on urine volume only. In addition, data were collected over two consecutive months and no washout period was allowed between the two months; and the KDIGO2012 guideline defined AKI as the SCr change over seven days period. Thus, some patients may had started developing AKI in the first month but did not need ER until the beginning of the following month. With that being said, the effect of no washout period would be limited to the first seven days of the following month, so we do not believe it would reverse our results or conclusion but future studies investigating this, or other similar objectives, should allow for a washout period between Ramadan and the following month.

## Conclusion

The results of this study showed a significant reduction in the incidence of AKI in the fasting cohort of patients as compared with a matched cohort of nonfasting individuals. Moreover, patients with additional risk factors for AKI were at lower risk of developing AKI in the fasting cohort than in nonfasting cohort. Thus, patients with an increased risk of AKI would not be harmed from fasting during Ramadan. Moreover, the benefit of fasting for patients with predisposing risk factors for AKI can be considered a nonpharmacologic intervention. Lastly, to have a better understanding for the effect of fasting on the risk of AKI in patients with comorbidities, large prospective studies are needed to confirm the findings of this study.

## Data Availability

The datasets used and/or analyzed during the current study are available in the supplementary files or from the corresponding author on request.
